# Supplementary material for: A survey of knowledge and attitudes towards antibiotic use and resistance among teachers in the Republic of Kenya: Implications for using teachers in raising public awareness of rational antibiotic use in school communities
Source: PLoS One. 2024 Dec 23;19(12):e0316122. doi: 10.1371/journal.pone.0316122 (PMC11666050; doi:10.1371/journal.pone.0316122)
Supplement: S1 Questionnaire — (DOCX) [file pone.0316122.s001.docx]

**Before you respond to the questionnaire items, you are requested to read and understand the attached consent form and Select YES if you consent and continue to respond to the questionnaire items. If you do not consent, kindly select NO and do not proceed to respond to the questionnaire items**

**INFORMED CONSENT FORM**

**Informed Consent**

My name is Patrick M. Mutua and I am the lead investigator of a cross-sectional study titled “A Survey of Knowledge and Attitude Towards Antibiotic use and Resistance among the Teachers in the Republic of Kenya: Implications for Using Teachers in Raising Public Awareness on Rational Antibiotic use in School Communities”

**Procedures to be followed**

Participation in this study will require that you respond to online questions related to your knowledge on and attitude towards Antibiotic use and Resistance. The study will provide baseline data that is critical in using teachers in improving school communities’ knowledge and attitudes for rational use of antibiotics.

**Voluntarism**

You have the right to refuse participation in this study and, therefore, participation in this study is voluntarily. You may ask questions related to the study at any time. You may refuse to respond to any questions and you may stop an interview at any time.

**Discomforts and Risks**

Some of the questions you will be asked are on intimate subject and may be embarrassing or make you uncomfortable. If this happens, you may refuse to answer these questions if you so choose. You may also stop the interview at any time.

**Benefits**

If you participate in this study, you will help us to gather information on teachers’ knowledge for and attitude toward proper antibiotic use and help in mitigating identified gaps for rational use of antibiotics within school communities.

**Reward**

Kindly be informed that participation in this study will not attract rewards or any payment to you.

**Confidentiality**

The online questionnaire is in form of google form and contains items that require you to respond to. Your name or personal data such as telephone number or email address will not be recorded on the questionnaire. Everything will be kept private and only shared with the study team.

**Contact Information**

If you have questions about the study, please contact the lead investigator Dr. Patrick M. Mutua using the email address [patmbuvi@gmail.com](mailto:patmbuvi@gmail.com)

**Participant’s statement**

The above information regarding my participation in the study is clear to me. The study has been explained to me and I have been given a chance to ask questions and my questions have been answered to my satisfaction. My participation in this study is entirely voluntary. I understand that my records will be kept private and that I can leave the study at any time. I, therefore, consent to participate in the study.

Please tick appropriately

Yes. **Please continue and complete the online questionnaire**

No. **Please stop the interview. Thank you for your time**

**Questionnaire**

1. **Socio-Demographic Information**

Participants have a right not to answer any question and may withdraw from the study at

any time. Please tick the option that is applicable to you. Some questions will also require

writing in the space provided.

**1.1 Age**

18 - 29

30 – 39

40 – 49

50 – and above

**1.2 Gender**

Male

Female

**1.3 Marital status**

Single

married

Widowed

Divorced

**1.4 Highest level of education achieved**

Certificate

Diploma

Degree

Post graduate education

**1.5** Select your sub county

**1.6** Type of school

Primary

Secondary

**1.7 Where do you get the antibiotics from?**

(a) From a pharmacy/Health center on prescription

(b) From a pharmacy without prescription

**1.8 Have you ever been exposed to any educational campaign on antibiotic resistance?**

Yes

No

Don’t know

2. **Information Assessing Knowledge about Antibiotics use and Antibiotic resistance**

Participants have a right not to answer any question and may withdraw from the study at

any time. Please tick one of the boxes for each statement to indicate your response.

**2. 1 I can differentiate between a bacterial and viral infection**

Yes

No

Don’t know

**2. 2 Viruses cause most cold and cough**

Yes

No

Don’t know

**2.3 Antibiotics are prescribed for most cough and cold**

Yes

No

Don’t know

**2.4 Antibiotics are effective for most sore throat**

Yes

No

Don’t know

**2.5 Antibiotics can kill bacteria**

Yes

No

Don’t know

**2.6 Antibiotics can kill viruses**

Yes

No

Don’t know

**2.7 Bacteria that live normally on the skin and in the guts are good for the health**

Yes

No

Don’t know

**2.8 Antibiotics do not kill the bacteria that live normally on the skin and in the gut**

Yes

No

Don’t know

**2.9 Antibiotics are the same as the medications used to relieve pain and fever such as**

**Aspirin and Tylenol**

Yes

No

Don’t know

**2.10 Antibiotic resistance means that bacteria will not be killed by antibiotics**

Yes

No

Don’t know

**2.11 Infections caused by antibiotic resistant bacteria cannot be easily cured or cannot be**

**cured**

Yes

No

Don’t know

**2.12 If antibiotics are taken for a long period of time, bacteria become resistant to**

**antibiotics**

Yes

No

Don’t know

**2.13 If antibiotics are taken less than the prescribed dose, bacteria become less resistant to**

**antibiotics**

Yes

No

Don’t know

**2.14 If twice the prescribed dose of antibiotics is taken, the effects of antibiotics are more**

**rapid**

Yes

No

Don’t know

**2.15 The prescribed dose and duration of antibiotics can be terminated if the symptoms**

**improve**

Yes

No

Don’t know

**2.16 Antibiotic resistance can spread between bacteria**

Yes

No

Don’t know

**2.17 Antibiotics have no side effect**

Yes

No

Don’t know

3. **Information Assessing Attitude to Antibiotic Use**

Participants have a right not to answer any question and may withdraw from the study at

any time. Please tick one of the boxes for each statement to indicate your response.

**3.1 I expect antibiotics to be prescribed by my doctor if I suffer from common cold**

**symptoms.**

Strongly agree

Agree

Neutral

Disagree

Strongly disagree

**3.2 If I catch a cold, I ask for an antibiotic prescription to prevent my symptoms from**

**getting worse**

Strongly agree

Agree

Neutral

Disagree

Strongly disagree

**3.3 I believe that antibiotics cure my cold faster**

Strongly agree

Agree

Neutral

Disagree

Strongly disagree

**3.4 I take left-over antibiotics when I have flu or other symptoms**

Strongly agree

Agree

Neutral

Disagree

Strongly disagree

**3.5 I stop taking the prescribed antibiotics once I get better**

Strongly agree

Agree

Neutral

Disagree

Strongly disagree

**3.6 I prefer a shot (Injection) to an oral medication if antibiotics are needed**

Strongly agree

Agree

Neutral

Disagree

Strongly disagree

**3.7 I check to see if antibiotics are included within the prescribed cold medicine**

Strongly agree

Agree

Neutral

Disagree

Strongly disagree

**3.8 I know which medication is an antibiotic when I take cold medicines**

Strongly agree

Agree

Neutral

Disagree

Strongly disagree

**3.9 If my family member is sick I usually give my prescribed antibiotic to them**

Strongly agree

Agree

Neutral

Disagree

Strongly disagree

**3.10 I normally keep antibiotic stock at home in case of emergency.**

Strongly agree

Agree

Neutral

Disagree

Strongly disagree

**3.11 I take antibiotics according to the instructions on the label.**

Strongly agree

Agree

Neutral

Disagree

Strongly disagree

**This is the end of the questionnaire, thank you for your time.**
