# Supplementary material for: A survey of knowledge and attitudes towards antibiotic use and resistance among teachers in the Republic of Kenya: Implications for using teachers in raising public awareness of rational antibiotic use in school communities
Source: PLoS One. 2024 Dec 23;19(12):e0316122. doi: 10.1371/journal.pone.0316122 (PMC11666050; doi:10.1371/journal.pone.0316122)
Supplement: Attachment — Submitted filename: RESPONSE TO REVIEWERS.docx [file pone.0316122.s002.docx]

From: Dr Patrick M. Mutua	To: The Editor

Department of Public Health, The PLoS ONE Journal

School of Health and Human Sciences,

Pwani University P.O Box 195-80108,

Kilifi, Kenya

26^th^ September, 2024

Email: [patmbuvi@gmail.com](mailto:patmbuvi@gmail.com)

Dear Sir/Madam

RE: REBUTTAL ON THE REVISED POINTS IN MY MANUSCRIPT PONE-D-24-18988

Thank you so much for you email dated 24^th^ September, 2024 requesting me to carry out revisions on our manuscript referenced above. My corrections are as follows:

To the academic Editor:

(1) I have carefully re-written references in line with PLOS one guidelines as advised.
(2) I have added more information in methods section indicating that consent was informed. Additionally, the draft of the questionnaire that was used to design the online questionnaire starts with consent information that requested participants to read and understand the informed consent and either agree on the statement before responding to the online questionnaire or disagree to the consent and therefore decline to proceed with the study. Kindly refer to the questionnaire submitted together with the revised manuscript
(3) In line with meeting the PLOS One journal requirement on availing data used in a study, I have submitted the questionnaire used to collect data as part of the attachment of the main manuscript. Kindly refer to the upload revised manuscript
(4) On sample selection, Under the Sample size section, I have included a statement on how we ensured data on sampled teachers was representative of the teachers by sub county and by school. The statement reads “In Kenya, there are 271 sub-counties. To ensure a representative sample of the teachers in the country, respondents were required to indicate their schools’ sub-county for random proportional distribution of the submitted questionnaires based on the number of teachers in each sub-county.” Additionally, the study involved all 271 Teachers Service Commission Sub County directors who distributed the online questionnaire to the teachers in the sub counties, providing opportunity for all teachers in all the sub counties to respond to the questionnaire.
(5) Under Methods and in the abstract, I have included Binary Logistic Regression as the model that was used to perform statistical analysis to determine the predicators of adequate knowledge and attitude toward antibiotic use and resistance.
(6) Under discussion section, I have included related studies in East Africa and Africa that included non-professionals in assessing knowledge for and attitude toward antibiotic use and resistance. I have further demonstrated how the findings of the related studies compare with the current study
(7) On Implications for policy and practice: In the conclusions section, the study recommends that policy makers in Kenya to use the study findings to plan comprehensive public education campaigns to mitigate gaps identified by the study so as to improve teachers’ knowledge on and attitude for antibiotic use. Further, the study recommends teacher training in colleges in universities to consider offering a course on AMR to teacher trainees. This will equip graduating teachers to have adequate knowledge and attitude on AMR and benefit school communities and the general public

To the Reviewers:

(1) I have carefully effected corrections to ensure all grammatical errors are corrected and I have highlighted the corrections for your perusal. The word goggle has been corrected to read google in line 137.
(2) Duplication of reference 15 and 24 has been corrected by deleting reference 24.
(3) References have been standardized based on PLOS journal guidelines
(4) On whether the question “I can differentiate between bacterial infections from Viral infections” is a knowledge or a perception question, we strongly feel the question is a knowledge one because it is assessing factual understanding. For one to respond to the question, they must have scientific knowledge to distinguish between bacterial and Viral infections based on their characteristics, causes, or symptoms. We, therefore, kindly request that the question be retained as it is in Table 2.
(5) We confirm that the questionnaire that we used in the study was previously used in another study in Nigeria. However, we carried out slight modifications to include a question on sub county of the respondent so as to ensure a representative sample of teachers in Kenya was well captured. We also removed a few items that sought history of use of antibiotics by respondents.

In conclusion, I believe that I have responded to all the corrections I have been requested to ensure are done and that my revised manuscript now meets all the PLoS ONE requirements for publication. I look forward to getting published in your esteemed journal. T

Regards

Dr Patrick M. Mutua
