# Supplementary material for: A survey of knowledge and attitudes towards antibiotic use and resistance among teachers in the Republic of Kenya: Implications for using teachers in raising public awareness of rational antibiotic use in school communities
Source: PLoS One. 2024 Dec 23;19(12):e0316122. doi: 10.1371/journal.pone.0316122 (PMC11666050; doi:10.1371/journal.pone.0316122)
Supplement: Attachment — Submitted filename: RESPONSE TO REVIEWERS.docx [file pone.0316122.s003.docx]

From: Dr Patrick M. Mutua	To: The Editor

Department of Public Health, The PLoS ONE Journal

School of Health and Human Sciences,

Pwani University P.O Box 195-80108,

Kilifi, Kenya

16^th^ November, 2024

Email: [patmbuvi@gmail.com](mailto:patmbuvi@gmail.com)

Dear Sir/Madam

RE: REBUTTAL ON THE REVISED POINTS IN MY MANUSCRIPT PONE-D-24-18988R1

Thank you so much for your email dated 15^th^ November, 2024 requesting me to carry out revisions on our manuscript referenced above. My corrections are as follows:

**To the academic Editor:**

(1) I have carefully checked all my references and I confirm that none of the references quoted in our manuscript is retracted

**To the Reviewers:**

**Corrections in the abstract**

(1) I have deleted the sentence “dearth of information…” and replaced this with “limited studies have investigated teachers’ knowledge of and attitudes towards antimicrobial use”
(2) In the abstract, I have clearly indicated the study focused on primary and secondary school teachers.
(3) With regard to use of knowledge, we have clearly indicated the knowledge score of teachers of antimicrobial resistance to be 9.2 out of 17.
(4) In the abstract, I have included the response rate of the participants as advised

**Corrections in main text**

(1) I have reduced the number of times I have mentioned “This is the first study to” and only mentioned this in the discussion as advised
(2) In our manuscript, we have quoted a related study done in Ghana that used storytelling to positively improve knowledge of antibiotics and antimicrobial resistance among children attending primary schools. This study involved primary school teachers. Therefore, it is possible for primary and secondary school teachers to teach about antimicrobial resistance using a variety of learner centered approaches as is the case with the Ghana study
(3) I have deleted the first four sentences in my conclusion as advised and the revised section starts with the findings
(4) I have used the word to MOTIVATE rather than to provoke…in the conclusions section with regard to using the findings to MOTIVATE teacher training colleges and universities to consider a course of antimicrobial resistance for teacher trainees

In conclusion, I believe that I have responded to all the corrections I have been requested to ensure are done and that my revised manuscript now meets all the PLoS ONE requirements for publication. I look forward to getting published in your esteemed journal. T

Regards

Dr Patrick M. Mutua
